# Supplementary material for: Relationships between radiation risk perception and health anxiety, and contribution of mindfulness to alleviating psychological distress after the Fukushima accident: Cross-sectional study using a path model
Source: PLoS One. 2020 Jul 6;15(7):e0235517. doi: 10.1371/journal.pone.0235517 (PMC7337340; doi:10.1371/journal.pone.0235517)
Supplement: S1 Fig — (a) Lower-educated in Fukushima, (b) Highly-educated in Fukushima, (c) Lower-educated in Tokyo, (d) Highly-educated in Tokyo. (DOCX) [file pone.0235517.s001.docx]

S1 Fig. Pass model results for stratification of region and education level.

1. Lower-educated in Fukushima


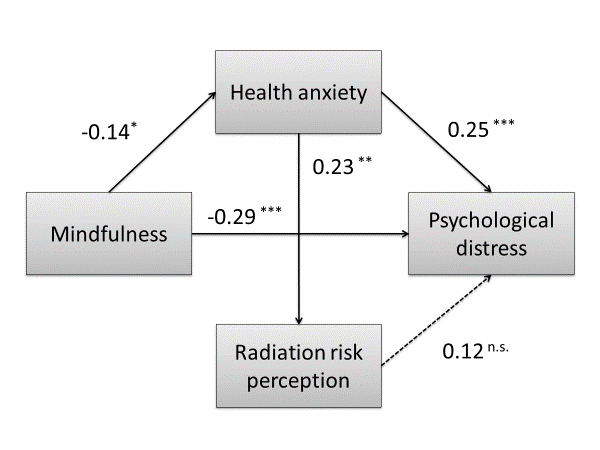


1. Highly-educated in Fukushima


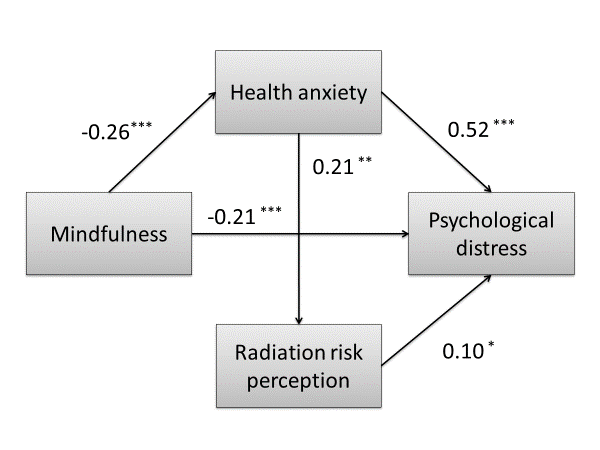


1. Lower-educated in Tokyo


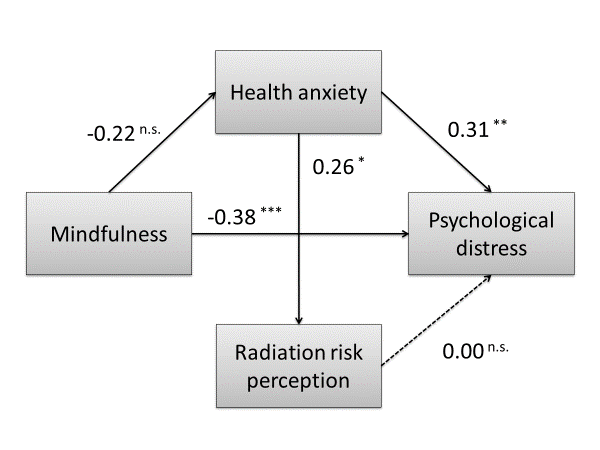


1. Highly-educated in Tokyo


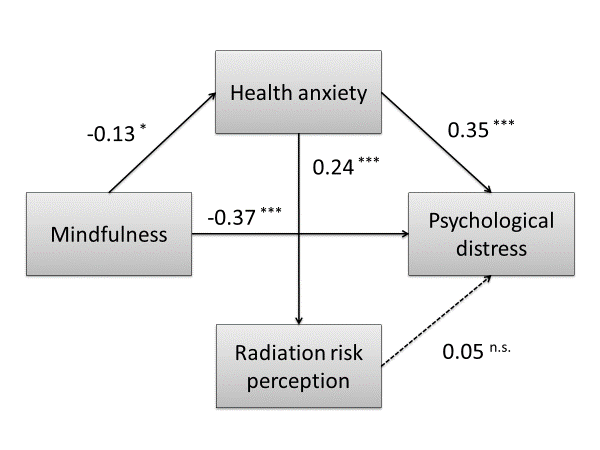


All the coefficients are standardized estimates, **P*<0.05, ***P*<0.01, ****P*<0.001.
